# Supplementary material for: The deubiquitylating enzyme Fat facets promotes Fat signalling and restricts tissue growth
Source: Nat Commun. 2025 Feb 24;16:1938. doi: 10.1038/s41467-025-57164-3 (PMC11850632; doi:10.1038/s41467-025-57164-3)

Supplementary Information

Supplementary Figure 1 – *In vivo* RNAi screens to identify Ft signalling regulators

Supplementary Figure 2 – Characterisation of morphological traits in adult wings

Supplementary Figure 3 – Ft protein levels in *ft* trans-heterozygous mutants

Supplementary Figure 4 – Genetic interactions between *faf* and Hpo and Ft pathway genes

Supplementary Figure 5 – Genetic interactions between *faf*, *hpo* and *wts*

Supplementary Figure 6 – Faf-mediated regulation of Ft protein levels

Supplementary Figure 7 – *In vivo* regulation of Ft protein levels

Supplementary Figure 8 –*In vivo* regulation of D subcellular localisation

Supplementary Figure 9 – Faf catalytic activity is required for its function in the regulation of Hpo signalling and tissue growth

Supplementary Figure 10 – Effect of proteasome and DUB inhibitors and conservation of Faf function in mammalian cells

Supplementary Table S1 – *In vivo* RNAi screen information

Supplementary Table S2 – Statistical analyses

Figure S6a Source Data

Figure S6d Source Data

Figure S10a Source Data

Figure S10b Source Data

Figure S10c Source Data

Figure S10h Source Data

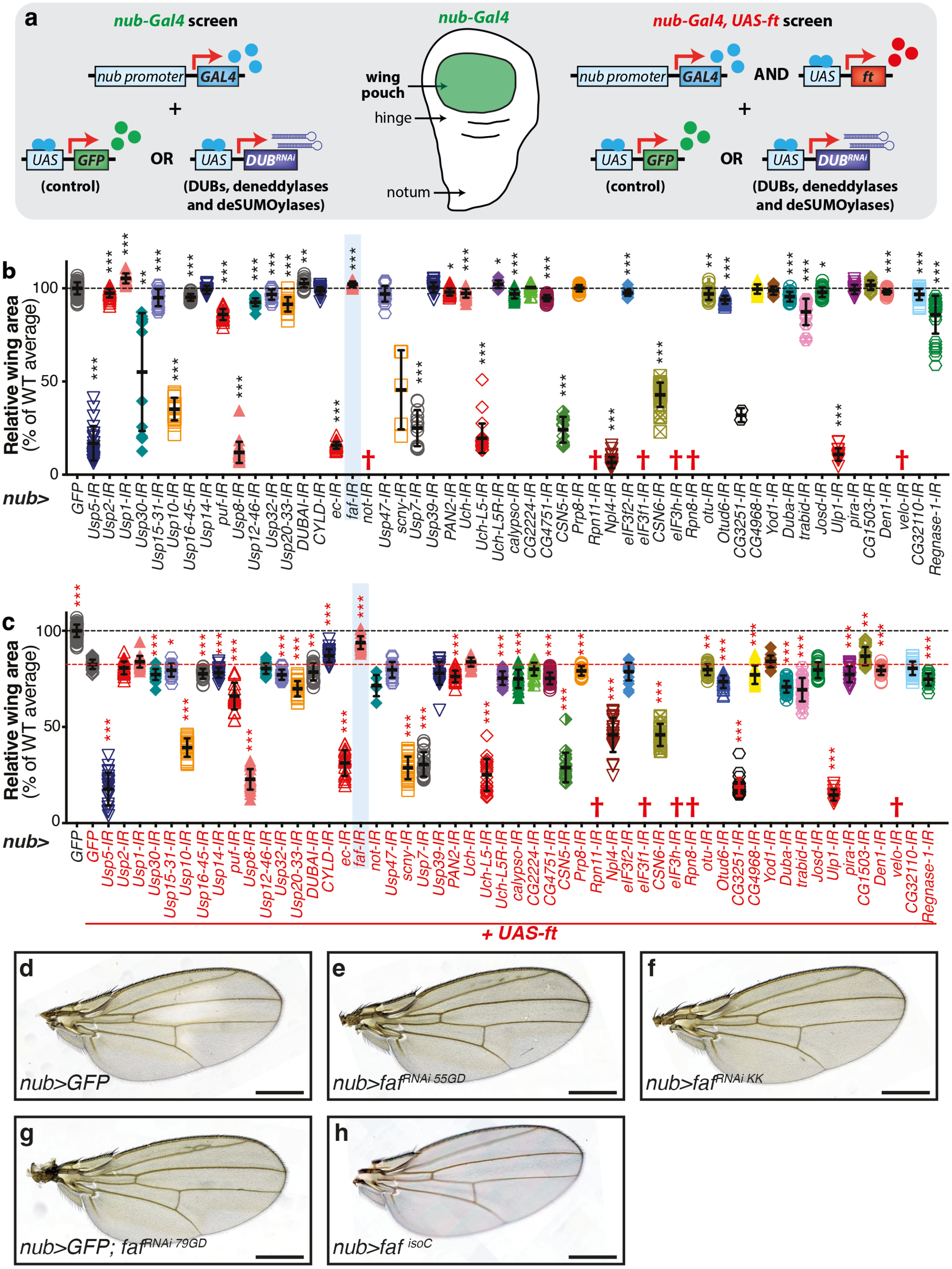

Supplementary Figure 1 – *In vivo* RNAi screens to identify Ft signalling regulators

(a) Schematic representation of the *in vivo* RNAi genetic screens performed. Temporal and spatial control of gene expression was achieved by using the *nub-Gal4* driver (*nub>*), such that, in the developing wing, expression of *UAS-RNAi* genes (targeting DUBs,

deneddylases and deSUMOylases) is limited to the pouch region. **(b, c)** Quantification of relative adult wing sizes from *nub-Gal4* (*nub>*; b) and *nub-Gal4, UAS-ft* (*nub>ft*; c) screens. Data are represented as % of the average wing area of the respective controls. (*nub>GFP*, average set to 100%). Data are shown as average ± standard deviation, with all data points depicted. Significance was assessed using Brown-Forsythe and Welch ANOVA analyses comparing all genotypes to the respective control, with Dunnett's multiple comparisons test. *, p<0.05; **, p<0.01; ***, p<0.001. Dashed black line indicates 100% (average of the *nub>GFP* control), while red dashed line indicates the average size of adult wings from *nub>ft, GFP* flies. † depicts instances where fly crosses resulted in lethality. **(d-g)** Validation of *faf* as a *nub-Gal4* screen hit. Shown are adult wings from flies raised at 25ºC expressing the indicated transgenes in the wing pouch under the control of *nub-Gal4*. Compared to control adult wings expressing GFP (d), depletion of *faf* in the developing wing resulted in increased tissue growth (e, f and g). **(h)** In contrast to faf^{RNAi}, *faf* over-expression resulted in an undergrowth phenotype. Scale bar represents 500 μm.

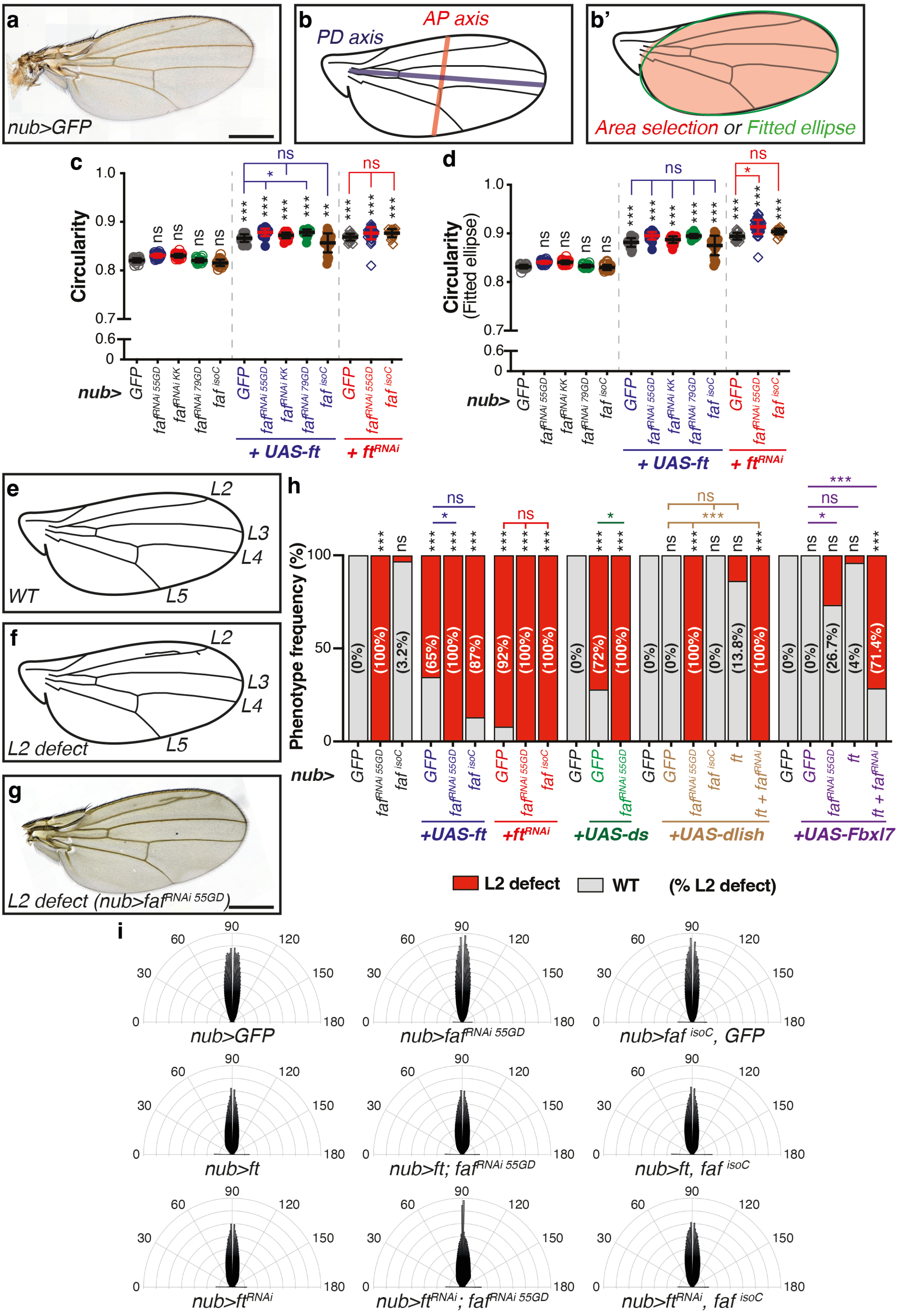

Supplementary Figure 2 – Characterisation of morphological traits in adult wings

(a) Representative image of control adult wing (*nub>GFP* female). **(b)** Schematic representation of the defining axes of the adult wing. AP: anterior-posterior; PD: proximal-distal. **(b')** Schematic representation of wing shape measurements, by analysis of a selected area of the adult wing (red area) or of a fitted ellipse of the wing area (green oval). **(c,d)** Quantification of wing shape. Data is represented as circularity measures of the area selection (c) or of a fitted ellipse of the wing area (d). All data is represented as average ± standard deviation, with all data points depicted. Vertical dashed lines separate different genotype conditions (*nub>*, *nub>ft* or *nub>*ft^{RNAi}). Significance was assessed using Kruskal-Wallis ANOVA analyses comparing all genotypes to the respective control (*nub>GFP, nub>ft* or *nub>*ft^{RNAi}; black, blue or red asterisks, respectively), with Dunn's multiple comparisons test. *, p<0.05; **, p<0.01; ***, p<0.001. ns, non-significant. (n= 22, 25, 29, 20, 31, 26, 27, 29, 25, 23, 25, 47, 19). **(e-g)** Scoring of morphological defects in wing vein pattern. (e) Schematic representation of control adult wing, with the normal pattern of venation. (f) Schematic representation of adult wing with a defect in L2. (g) Representative example of an adult wing with L2 defect (*nub-Gal4, UAS-*$faf^{RNAi\ 55GD}$ female). Scale bar represents 500 μm. **(h)** Frequency of L2 defects. Shown are the frequencies of adult wings with (red) or without a defect in the L2 vein (grey) in the indicated genotypes. Significance was calculated using Chi-square analysis, adjusted Fisher's exact test and Bonferroni correction for multiple comparisons. *, p<0.05; **, p<0.01; ***, p<0.001. ns, non-significant. (n= 23, 25, 31, 26, 27, 23, 25, 47, 19, 23, 25, 30, 20, 28, 26, 19, 29, 28, 23, 28, 30, 25, 28). **(i)** Analysis of wing hair orientation. Shown are 180º rose plots indicating orientation angles of wing hairs in regions of interest defined in the region between the L3 and L4 wing veins for the indicated genotypes, with 0º corresponding to the vertical direction and 90º to the horizontal direction. (n=30 ROIs for all genotypes except *nub-Gal4*, where n=33).

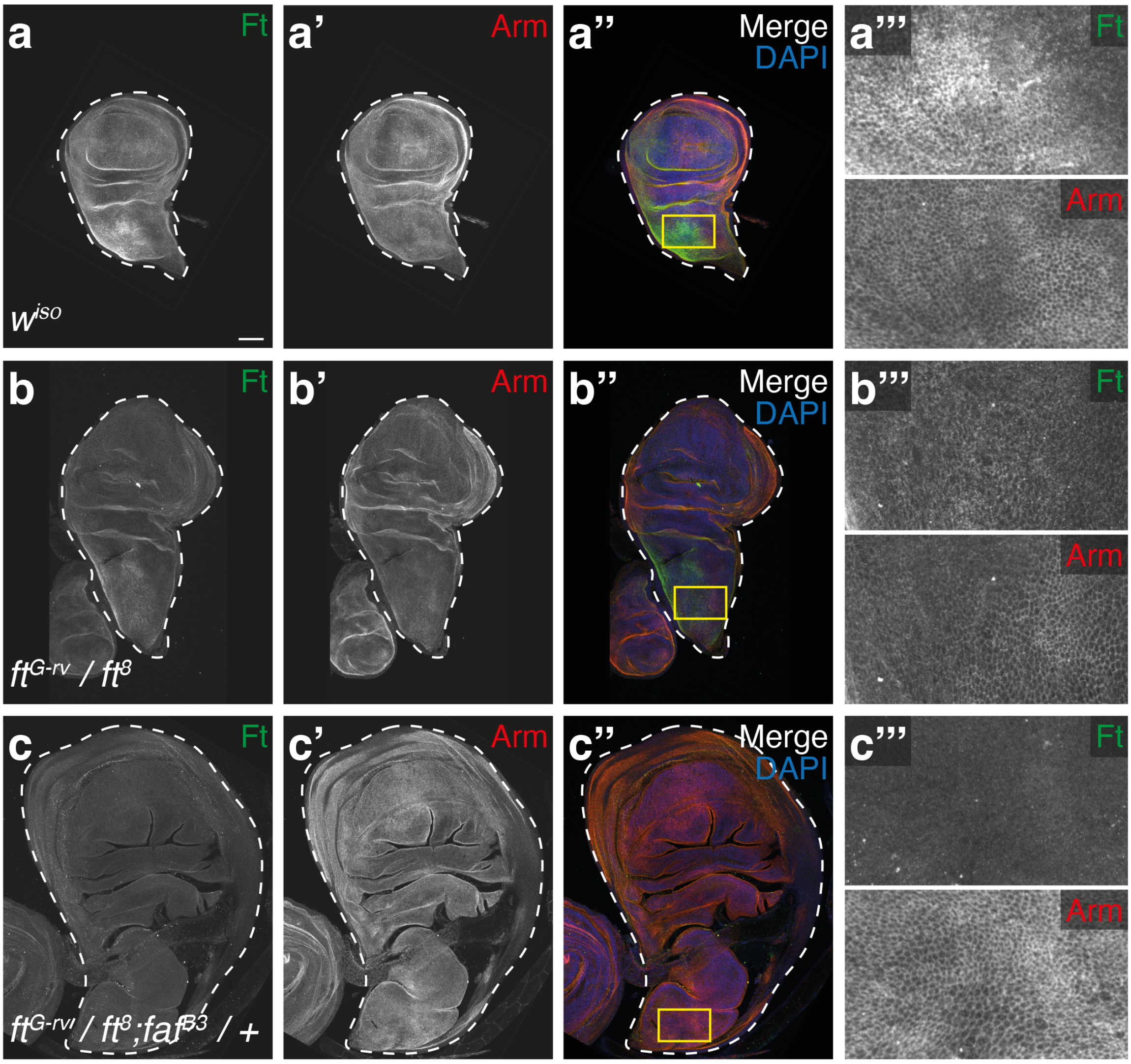

Supplementary Figure 3 – Ft protein levels in *ft* trans-heterozygous mutants

(a-c) Confocal micrographs of third instar wing imaginal discs from the indicated genotypes showing Ft (a-c, a’’’-c’’’ and green in a’’-c’’ merged images) and Arm antibody staining (a’-c’, a’’’-c’’’ and red in a’’-c’’ merged images). DAPI (blue) stains nuclei. Yellow boxes indicate area of the wing disc shown in inset images (a’’’-c’’’). Scale bar represents 50 μm.

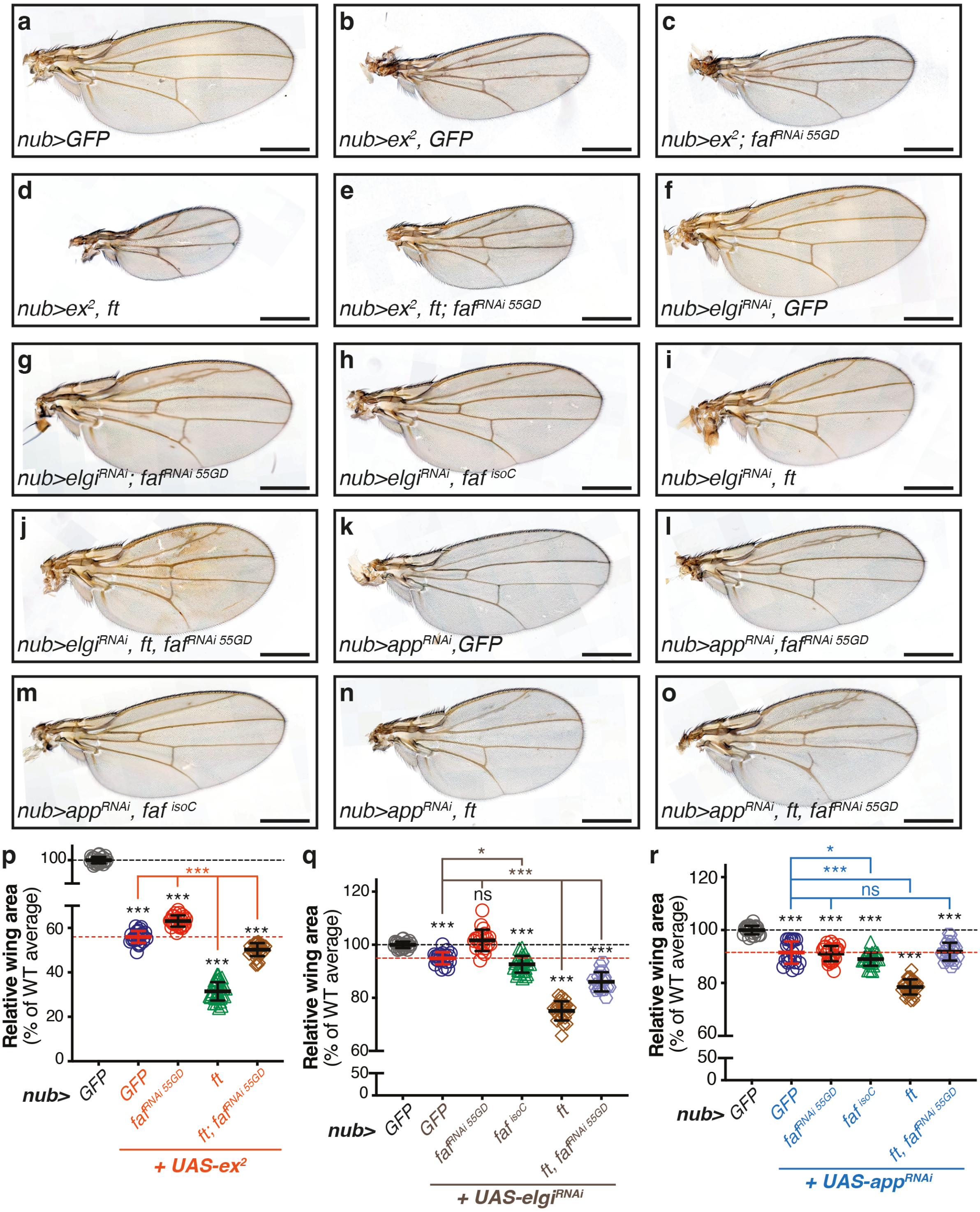

Supplementary Figure 4 – Genetic interactions between *faf* and Hpo and Ft pathway genes

(a-o) Genetic interactions between *faf* and *ex* (a-e), *elgi* (f-j) or *app* (k-o). Shown are adult wings from flies raised at 25ºC expressing the indicated transgenes in the wing pouch under the control of *nub-Gal4* (*nub*>). Over-expression of ex^2 (mild *ex* allele) under the control of *UAS* sequences resulted in a dramatic decrease in wing size (b) compared to

WT wings (a), which was partially suppressed by *faf* depletion (c). In contrast, expression of *ft* enhanced the ex^2 undergrowth phenotype (d), and this was counteracted by depletion of *faf* (e). Depletion of *elgi* ($elgi^{RNAi}$) resulted in a mild decrease in tissue growth (f), which was abrogated when *faf* was co-depleted (g). Over-expression of either *faf* (h) or *ft* (i) enhanced the undergrowth phenotype of $elgi^{RNAi}$-expressing flies and the *ft* size phenotype was rescued by faf^{RNAi} (j). Depletion of *app* (app^{RNAi}) resulted in a mild decrease in tissue growth (k), which was not affected by modulation of *faf* levels (l, m). Expression of *ft* enhanced the undergrowth phenotype (n) and this was rescued by depletion of *faf* (o). **(p-r)** Quantification of relative adult wing sizes. Data are represented as % of the average wing area of control wings (*nub>GFP*, set as 100%). Data are shown as average ± standard deviation, with all data points represented. (n=23, 26, 25, 31, 28 for p; n=23, 22, 21, 26, 24, 19 for q; and n=22, 22, 25, 23, 27, 24 for r). Black dashed lines represent average size of controls (100%), whilst red dashed lines indicate average size of adult wings from flies expressing ex^2, $elgi^{RNAi}$ or app^{RNAi} under the control of *nub-Gal4*. Significance was assessed using a one-way ANOVA comparing all genotypes to their respective controls (*nub>GFP*, *nub>UAS-*ex^2, *nub>UAS-*$elgi^{RNAi}$, *nub>UAS-*app^{RNAi}, black, orange, brown and blue asterisks, respectively), with Dunnett's multiple comparisons test. *, p<0.05; **, p<0.01; ***, p<0.001; ns, non-significant. Scale bar represents 500 μm.

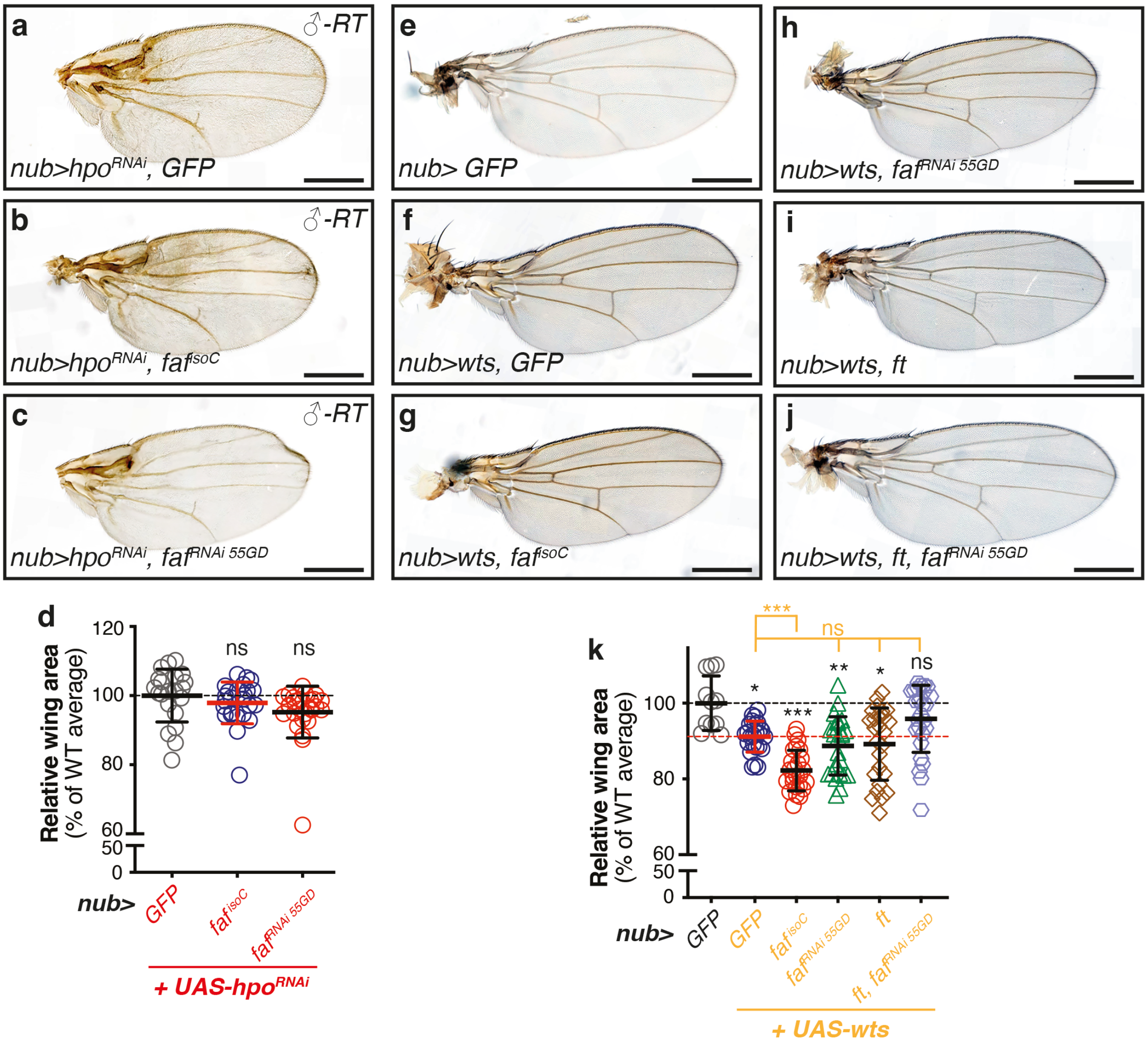

Supplementary Figure 5 – Genetic interactions between *faf*, *hpo* and *wts*

(a-c) Modulation of *faf* expression does not alter growth phenotypes elicited by *hpo* RNAi-mediated depletion. Shown are adult wings from male flies raised at room temperature (RT) expressing the indicated transgenes in the wing pouch using *nub-Gal4* (*nub>*). Crosses were raised at RT due to lethality at 25ºC. Depletion of *hpo* results in wing overgrowth (a). Expression of faf^{isoC} (b) or *faf* depletion (c, $faf^{RNAi\ 55GD}$) did not alter the phenotype of Hpo-depleted wings. **(d)** Quantification of relative adult wing sizes in hpo^{RNAi} experiment. Data are represented as % of the average wing area of control wings (*nub>GFP, UAS-*hpo^{RNAi}, set as 100%.). Data are shown as average ± standard deviation, with all data points represented. (n=21, 25, 27). Black dashed line represents average size of controls (100%). Significance was assessed using a Brown-Forsythe and

Welch ANOVA comparing all genotypes to the control, with Dunnett's multiple comparisons test. ns, non-significant. Scale bar represents 500 μm. **(e-j)** Modulation of *faf* levels has minimal effect on *wts*-mediated growth phenotypes. Shown are adult wings from flies raised at 25ºC expressing the indicated transgenes in the wing pouch under the control of *nub-Gal4* (*nub*>). *wts* over-expression of *wts* resulted in undergrowth (f) compared to WT wings (e), which was enhanced by faf^{isoC} expression (g). In contrast, *faf* depletion (h), *ft* expression (i) or co-expression of *ft* and faf^{RNAi} (j) had no effect on the *wts* phenotype. **(k)** Quantification of relative adult wing sizes in *wts* experiments. Data are represented as % of the average wing area of control wings (*nub>GFP*, set as 100%). Data are shown as average ± standard deviation, with all data points represented. (n=11, 25, 25, 24, 26, 27). Black dashed lines represent average size of controls (100%), whilst red dashed line indicates average size of adult wings from flies expressing *wts*, under the control of *nub-Gal4*. Significance was assessed using a one-way ANOVA comparing all genotypes to their respective controls (*nub>GFP*, *nub>UAS-wts*, black and orange asterisks, respectively), with Dunnett's multiple comparisons test. *, $p<0.05$; **, $p<0.01$; ***, $p<0.001$; ns, non-significant. Scale bar represents 500 μm.

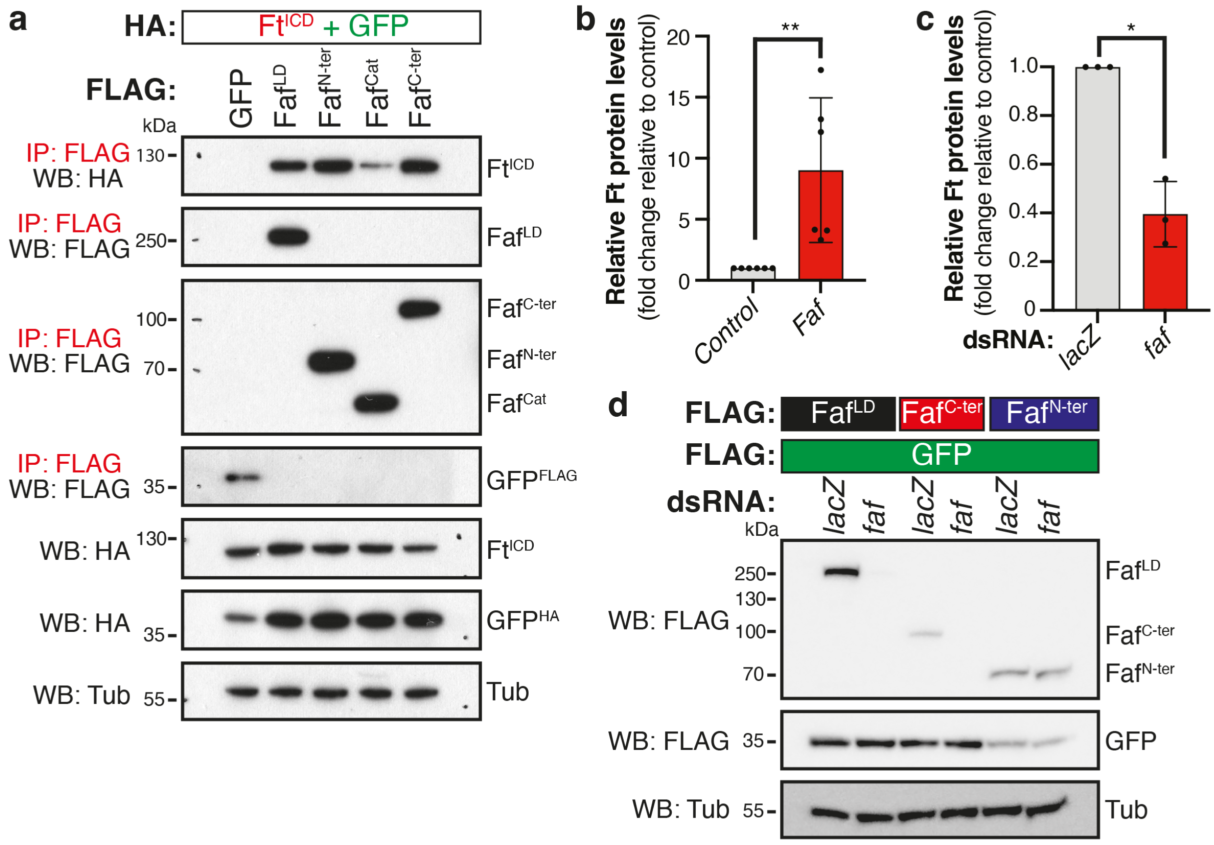

Supplementary Figure 6 – Faf-mediated regulation of Ft protein levels

(a) Mapping interaction between Faf and Ft. HA-tagged Ft^{ICD} and GFP were co-expressed with FLAG-tagged GFP, Faf^{LD} or Faf truncations in *Drosophila* S2 cells and lysates were analysed by immunoblot using the indicated antibodies following FLAG co-immunoprecipitation. HA-tagged GFP and Tubulin (Tub) were used as transfection and loading controls, respectively. **(b,c)** Quantification of effect of Faf modulation in S2 cells on Ft protein levels. Shown are the relative Ft protein levels (fold change relative to controls, set to 1; Ft protein levels were normalised to Tubulin) quantified from Western blot experiments where Faf was overexpressed (b) or depleted (c). Data are represented as average ± standard deviation, with all data points represented. n=6 (b) or n=3 (c) independent experiments. Significance was assessed by unpaired two-tailed t-test with Welch's correction. *, $p<0.05$ **(d)** Validation of *faf* RNAi efficiency. RNAi specificity and efficiency were analysed by Western blotting using the indicated antibodies. Note that *faf* dsRNA targeting sequences overlap with Faf^{LD} and $Faf^{C\text{-}ter}$, but not with the $Faf^{N\text{-}ter}$

construct and, as expected, only the latter was unaffected by the dsRNA. FLAG-tagged GFP and Tubulin (Tub) were used as transfection and loading controls, respectively.

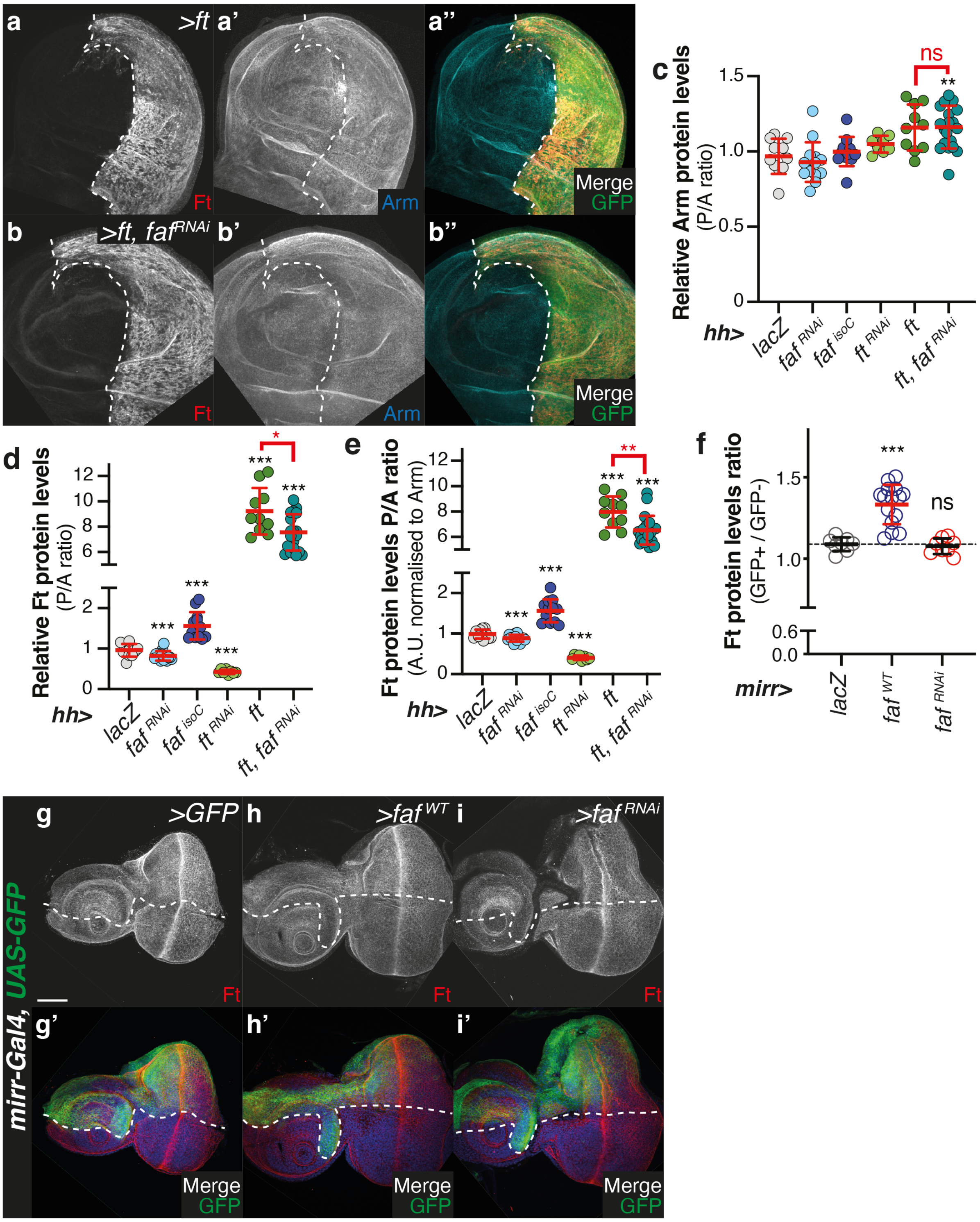

Supplementary Figure 7 – *In vivo* regulation of Ft protein levels

(a,b) Faf regulates Ft protein levels *in vivo*. Shown are XY confocal micrographs of third instar wing imaginal discs expressing the indicated constructs under the control of *hh-Gal4*, showing Ft antibody staining (a, b; red in a'', b''), Arm antibody staining (a', b'; cyan in a'', b''), and direct fluorescence from GFP (green in a'', b''). Ventral is up in XY sections,

whilst GFP marks the *hh-Gal4*-expressing posterior compartment (right). Dashed white line depicts boundary between anterior and posterior compartments. (n=10, 20). Scale bar: 50 μm. **(c-e)** Quantification of *in vivo* Arm and Ft protein levels in the genotypes from Figures 4 and S7. Shown are the posterior/anterior (P/A) ratios for Arm (c), or Fat protein levels (d), as well as the normalised Ft protein levels (e, normalised to Arm). Data are shown as average ± standard deviation, with all data points represented. (n=11, 14, 12, 8, 10, 20). Significance was assessed using a Brown-Forsythe and Welch ANOVA comparing all genotypes to their respective controls, with Dunnett's multiple comparisons test. Significance of pairwise comparisons was assessed using an unpaired two-tailed t-test, with Welch's correction. *, $p<0.05$; **, $p<0.01$; ***, $p<0.001$; ns, non-significant. **(f-i)** Faf-mediated regulation of Ft protein levels in the eye imaginal disc. (f) Quantification of relative Ft protein levels. Data are shown as average ± standard deviation, with all data points represented. (n=8, 15, 8). Significance was assessed using an unpaired two-tailed t-test, with Welch's correction. ***, $p<0.001$; ns, non-significant. (g-i) XY confocal images of third instar eye imaginal discs expressing the indicated constructs under the control of *mirr-Gal4*, showing Ft antibody staining (g-i; red in g'-i') and direct GFP fluorescence (green in g'-i'). DAPI (blue) marks nuclei. GFP marks the *mirr-Gal4* domain. Scale bar represents 50 μm.

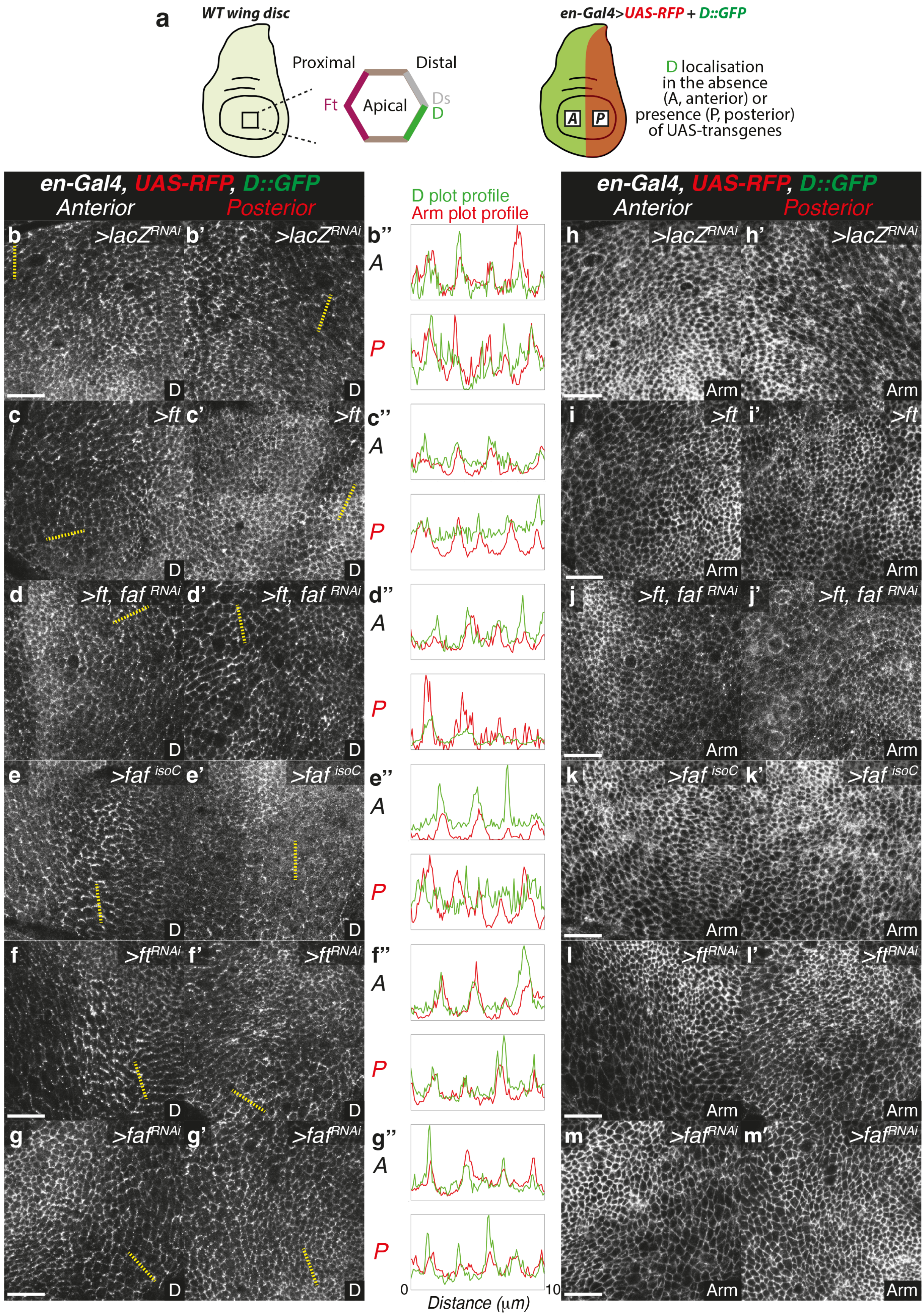

Supplementary Figure 8 –*In vivo* regulation of D subcellular localisation

(a) Schematic representation of subcellular localisation of Ft signalling components and experimental setting. D localisation was monitored using a D::GFP transgene, whilst expression of genes of interest was controlled by *en-Gal4*, which is expressed in the posterior compartment of the wing imaginal disc (marked by RFP expression). **(b-g)** Regulation of D subcellular localisation by Ft and Faf. XY sections of third instar wing imaginal discs, showing direct fluorescence from GFP in the control anterior compartment (b-g) or experimental posterior compartment (b'-g'). (b''-g'') Shown are the plot profiles for D (green) and Arm staining (red) in the anterior (A, top panels) or posterior (P, bottom panels) compartments (10 μm profile; yellow dashed lines). Cartoons represent expected results for each genotype tested. Over-expression of *ft* or *faf* results in mislocalisation of D, which appears more cytoplasmic (c' and e'), compared to controls (b'). The *ft* over-expression phenotype was largely rescued by co-depletion of *faf* (d'). (n=13, 7, 13, 8, 6, 13). Scale bar: 10 μm. **(h-m)** Effect of *ft* and *faf* depletion on Arm subcellular localisation. XY sections of third instar wing imaginal discs, showing Arm staining in the control anterior compartment (h-m) or experimental posterior compartment (h'-m'). No overt effects were observed for Arm in all genotypes tested. (n=13, 7, 13, 8, 6, 13). Scale bar: 10 μm.

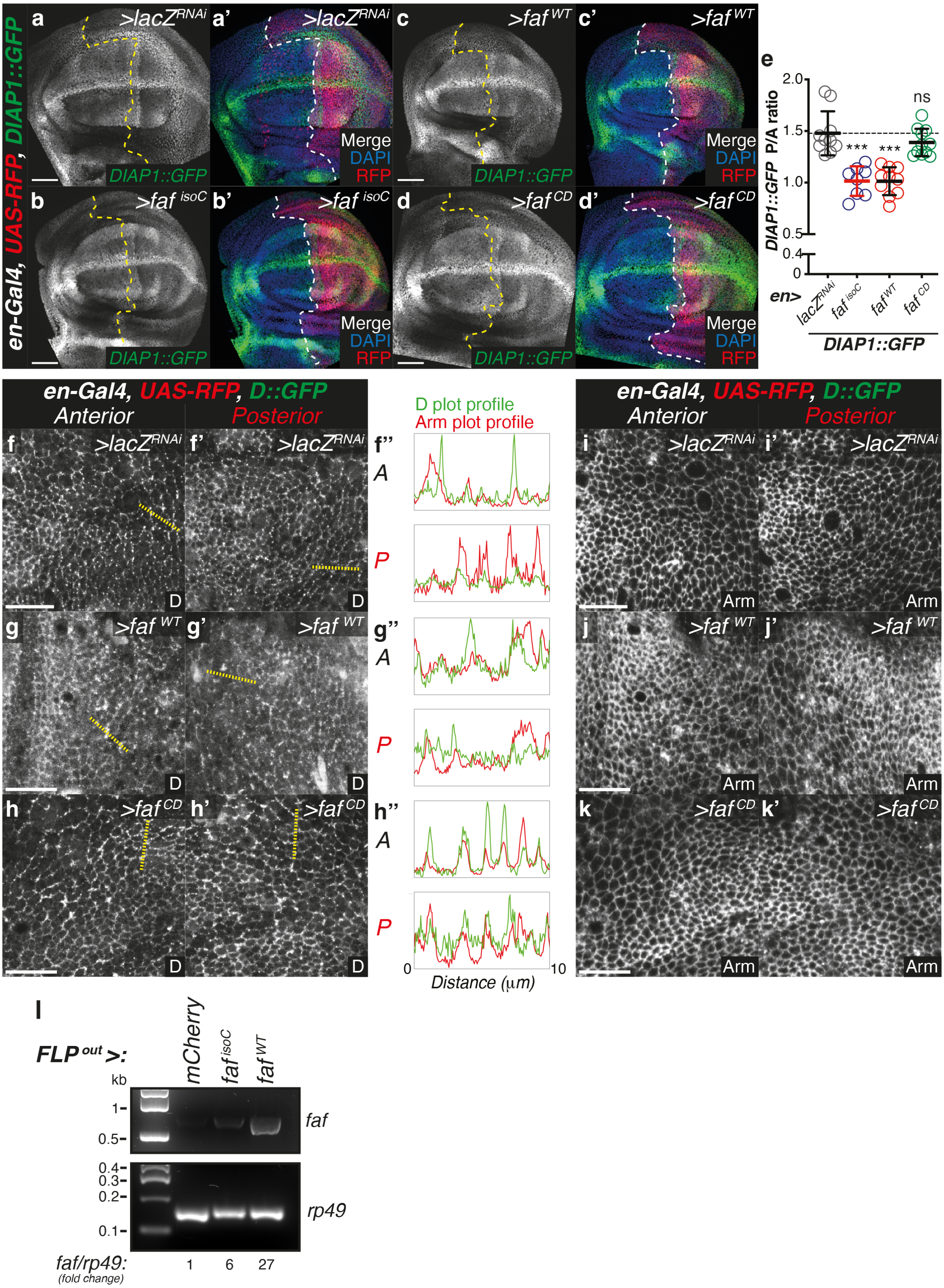

Supplementary Figure 9 – Faf catalytic activity is required for its function in the regulation of Hpo signalling and tissue growth

(a-d) Faf-mediated regulation of Yki target gene expression is dependent on its catalytic activity. XY confocal sections of third instar wing imaginal discs carrying *DIAP1::GFP* (a-d; green in a'-d'), in which *en-Gal4* was used to drive expression of *UAS-lacZ*^{RNAi} (a), *UAS-faf*^{isoC} (b), *UAS-faf*^{WT} (c) or *UAS-faf*^{CD} (d). RFP (red in a'-d') indicates posterior compartment where transgenes are expressed. DAPI (blue) stains nuclei. Dashed lines indicate anterior-posterior compartment boundary. **(e)** Quantification of *DIAP1::GFP* expression levels. Shown are posterior/anterior (P/A) *DIAP1::GFP* ratios. Data are shown as average ± standard deviation, with all data points represented. (n=10, 8, 10, 11). Significance was assessed using a one-way ANOVA comparing all genotypes to *UAS-lacZ*^{RNAi}, with Dunnett's multiple comparisons test. ***, $p<0.001$; ns, non-significant. Scale bar represents 50 μm. **(f-h)** Effect of Faf catalytic activity on regulation of D localisation. XY sections of third instar wing imaginal discs, showing direct fluorescence from GFP in the control anterior compartment (f-h) or experimental posterior compartment (f'-h'). (f''-h'') Shown are the plot profiles for D (green) and Arm staining (red) in the anterior (A, top panels) or posterior (P, bottom panels) compartments (10 μm profile; yellow dashed lines). Scale bar: 10 μm. **(i-k)** Effect of Faf catalytic activity on Arm subcellular localisation. XY sections of third instar wing imaginal discs, showing Arm staining in the anterior (i-k) or posterior compartment (i'-k'). Scale bar represents 10 μm. **(l)** Analysis of expression levels of *faf* transgenes. Total RNA was extracted from flies of the indicated genotypes. cDNA was generated and used as template for PCR analysis using primers specific for *faf* or *rp49* (control). Relative expression of *faf* was calculated as fold change relative to *rp49* levels, which was set as 1 for controls (*mCherry*).

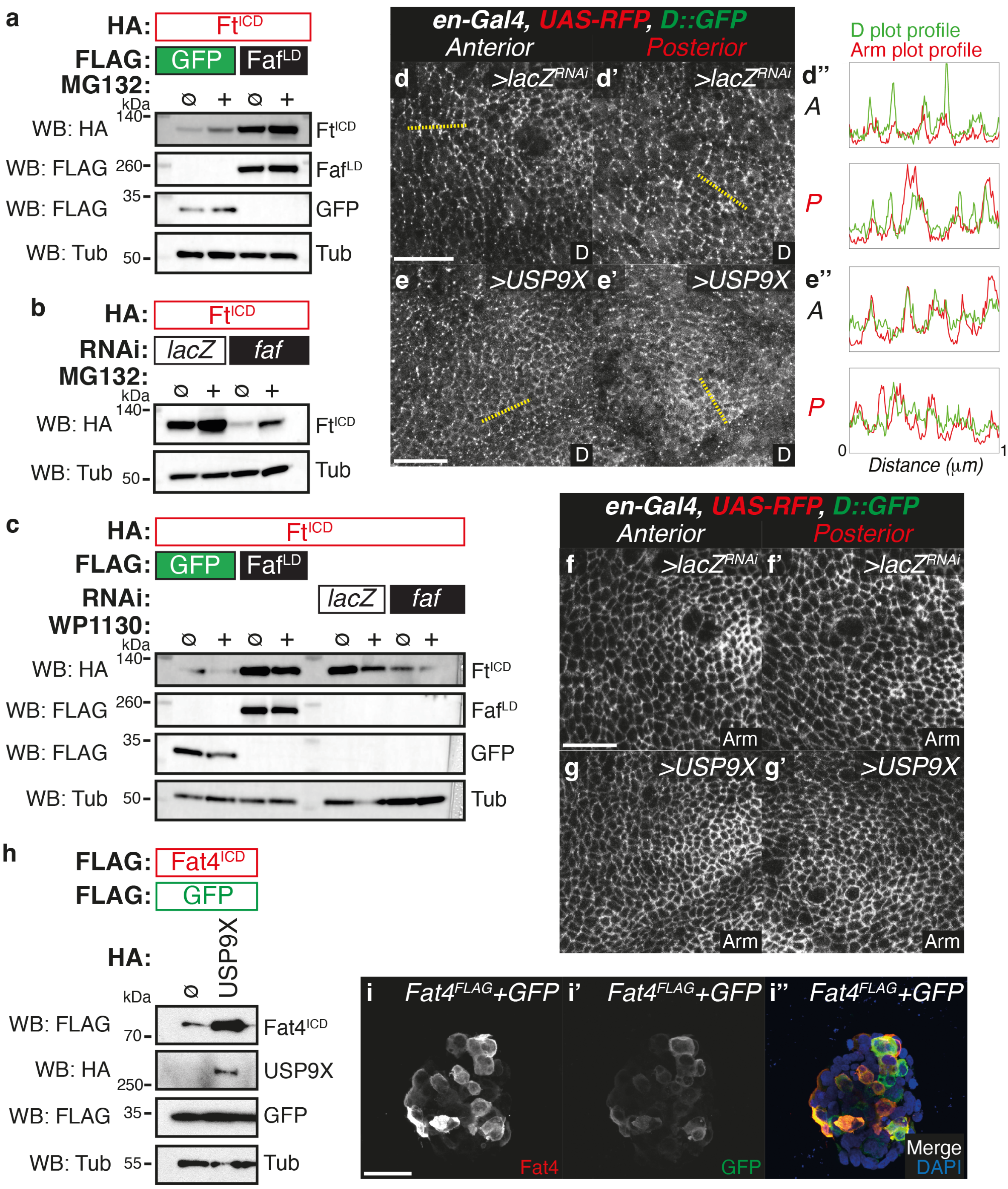

Supplementary Figure 10 – Effect of proteasome and DUB inhibitors and conservation of Faf function in mammalian cells

(a,b) Ft protein levels are regulated by the proteasome. (a) HA-tagged Ft^{ICD} was co-expressed with FLAG-tagged GFP or Faf^{LD} in *Drosophila* S2 cells. Cells were treated with MG132 prior to lysis and immunoblot analysis using the indicated antibodies. Compared to vehicle-treated samples (ø), MG132-treated cells (+) display higher levels of Ft. (b) S2 cells were treated with the indicated dsRNAs 24h prior to transfection with HA-tagged

Ft^{ICD}. Cells were treated with vehicle (ø) or MG132 (+) prior to lysis and immunoblot analysis with the indicated antibodies. **(c)** Effect of DUB inhibition on Ft protein levels. HA-tagged Ft^{ICD} was co-expressed with FLAG-tagged GFP or Faf^{LD}, or expressed 24h after treatment with the indicated dsRNAs in *Drosophila* S2 cells. Cells were treated with vehicle (ø) or WP1130 (+) prior to cell lysis and Western blot analysis with the indicated antibodies. Tubulin (Tub) was used as loading control. **(d, e)** USP9X regulates D subcellular localisation. XY sections of third instar wing imaginal discs, showing direct GFP fluorescence in the control anterior compartment (d, e) or experimental posterior compartment (d', e'). (d'', e'') Plot profiles for D (green) and Arm staining (red) in the anterior (A, top) or posterior (P, bottom) compartments (10 μm profile; yellow dashed lines). Scale bar: 10 μm. **(f, g)** Effect of USP9X on Arm subcellular localisation. XY sections of third instar wing imaginal discs, showing Arm staining in the anterior (f, g) or posterior compartment (f', g'). No overt effects were observed for Arm in all genotypes tested. Scale bar: 10 μm. **(h)** USP9X regulates $Fat4^{ICD}$ protein levels. FLAG-tagged $Fat4^{ICD}$ was co-expressed with FLAG-tagged GFP and either empty vector (ø) or HA-tagged USP9X in HEK293 cells. Lysates were analysed by immunoblot using the indicated antibodies. FLAG-GFP and Tubulin were used as transfection and loading controls, respectively. **(i)** Validation of Fat4 antibody in HEK293 cells. HEK293 cells were transfected with FLAG-tagged Fat4 and GFP, plated in coverslips and stained with Fat4 antibody and DAPI. Shown are XY confocal sections of transfected HEK293 cells depicting Fat4 antibody staining (i; red in i'') or direct GFP fluorescence (g'; green in i''). (i'') Merged image depicting Fat4 and GFP signals, as well as DAPI (blue) staining nuclei.

Table S1 – *In vivo* RNAi screen information

DUB family	CG#	Gene name	RNAi line
USP	*CG12082*	*Usp5*	NIG 12082R-2
USP	*CG14619*	*Usp2*	VDRC 104382 KK
USP	*CG15817*	*Usp1*	VDRC 41605 GD
USP	*CG3016*	*Usp30*	VDRC 7090 GD
USP	*CG30421*	*Usp15-31*	VDRC 33726 GD
USP	*CG32479*	*Usp10*	VDRC 37859 GD
USP	*CG4165*	*Usp16-45*	VDRC 110286 KK
USP	*CG5384*	*Usp14*	VDRC 110227 KK
USP	*CG5794*	*puffyeye (puf)*	VDRC 27517 GD
USP	*CG5798*	*Usp8*	VDRC 107623 KK
USP	*CG7023*	*Usp12-46*	VDRC 100586 KK
USP	*CG8334*	*Usp32*	VDRC 18981 GD
USP	*CG8494*	*Usp20-33*	VDRC 42609 GD
USP	*CG8830*	*DUBAI*	VDRC 28960 GD
USP	*CG5603*	*CYLD*	VDRC 15340 GD
USP	*CG2904*	*echinus (ec)*	VDRC 106671 KK
USP	*CG1945*	*fat facets (faf)*	VDRC 30679 GD
USP	*CG4166*	*non-stop (not)*	VDRC 45775 GD
USP	*CG5486*	*Usp47*	VDRC 26027 GD
USP	*CG5505*	*scrawny (scny)*	VDRC 105989 KK
USP	*CG1490*	*Usp7*	VDRC 110324 KK
USP	*CG7288*	*Usp39*	NIG 7288R-1
USP	*CG8232*	*PAN2*	NIG 8232R-1
UCH	*CG4265*	*Uch*	VDRC 103614 KK
UCH	*CG3431*	*Uch-L5*	VDRC 103481 KK
UCH	*CG1950*	*Uch-L5R*	NIG 1950R-1
UCH	*CG8445*	*calypso (caly)*	VDRC 47743 GD
JAMM	*CG2224*	*CG2224*	VRDC 108622 KK
JAMM	*CG4751*	*CG4751*	VDRC 45530 GD
JAMM	*CG14884*	*CSN5*	NIG 14884-R1
JAMM	*CG8877*	*Prp8*	VDRC 18565 GD
JAMM	*CG18174*	*Rpn11*	VDRC 19272 GD
JAMM	*CG4673*	*Npl4*	CG4673R-2
JAMM	*CG8335*	*eIF3f2*	VDRC 108169 KK
JAMM	*CG9769*	*eIF3f1*	VDRC 101465 KK
JAMM	*CG6932*	*CSN6*	VDRC 105385 KK
JAMM	*CG9124*	*eIF3h*	VDRC 36087 GD
JAMM	*CG3416*	*Rpn8*	VDRC 26183 GD
OTU	*CG12743*	*otu*	VDRC 108845 KK
OTU	*CG7857*	*Otud6*	NIG 7857R-2
OTU	*CG3251*	*CG3251*	VDRC 100532 KK
OTU	*CG4968*	*CG4968*	VDRC 21978 GD
OTU	*CG4603*	*Yod1*	VDRC 21893 GD
OTU	*CG6091*	*Duba*	VDRC 109912 KK
OTU	*CG9448*	*trabid (trbd)*	VDRC 24030 GD
JOSEPHIN	*CG3781*	*Josd*	VDRC 108379 KK
ULP	*CG12359*	*Ulp1*	VDRC 106625 KK
ULP	*CG12717*	*pirate (pira)*	VDRC 106239 KK
ULP	*CG1503*	*CG1503*	VDRC 32349 GD
ULP	*CG8493*	*Den1*	VDRC 100591 KK
ULP	*CG10107*	*veloren (velo)*	VDRC 103524 KK
ULP	*CG32110*	*CG32110*	VDRC 107634 KK
MCPIP	*CG10889*	*Regnase-1*	NIG 10889R-2

List of genes and RNAi lines tested in the *in vivo* RNAi screen. Shown are the DUB families, CG number, gene names and RNAi fly stock information. USP, UCH, JAMM,

OTU, JOSEPHIN, ULP and MCPIP denote ubiquitin-specific protease, ubiquitin C-terminal hydrolase, Jab1/Mov34/Mpr1 Pad1 N-terminal+ domain metalloprotease, ovarian tumour, Machado-Josephin domain, ubiquitin-like specific proteases and Monocyte Chemotactic Protein-Induced Protein, respectively.

Table S2 – Statistical analyses

Figure	Comparison (control group)	Comparison (experimental group)	p-value and significance value
1j	*nub-Gal4, UAS-GFP*	*nub-Gal4, UAS-faf* $^{RNAi\ 55GD}$	>0.9999; ns
1j	*nub-Gal4, UAS-GFP*	*nub-Gal4, UAS-faf* $^{RNAi\ KK}$	0.0006; ***
1j	*nub-Gal4, UAS-GFP*	*nub-Gal4, UAS-faf* $^{RNAi\ 79GD}$	0.7855; ns
1j	*nub-Gal4, UAS-GFP*	*nub-Gal4, UAS-faf* ^{isoC}	<0.0001; ***
1j	*nub-Gal4, UAS-GFP*	*nub-Gal4, UAS-ft*	<0.0001; ***
1j	*nub-Gal4, UAS-GFP*	*nub-Gal4, UAS-ft, UAS-faf* $^{RNAi\ 55GD}$	<0.0001; ***
1j	*nub-Gal4, UAS-GFP*	*nub-Gal4, UAS-ft, UAS-faf* $^{RNAi\ KK}$	<0.0001; ***
1j	*nub-Gal4, UAS-GFP*	*nub-Gal4, UAS-ft, UAS-faf* $^{RNAi\ 79GD}$	<0.0001; ***
1j	*nub-Gal4, UAS-GFP*	*nub-Gal4, UAS-ft, UAS-faf* ^{isoC}	<0.0001; ***
1j	*nub-Gal4, UAS-GFP*	*nub-Gal4, UAS-ft* ^{RNAi}	<0.0001; ***
1j	*nub-Gal4, UAS-GFP*	*nub-Gal4, UAS-ft* ^{RNAi}*, UAS-faf* $^{RNAi\ 55GD}$	<0.0001; ***
1j	*nub-Gal4, UAS-GFP*	*nub-Gal4, UAS-ft* ^{RNAi}*, UAS-faf* ^{isoC}	0.001; **
1j	*nub-Gal4, UAS-ft, UAS-GFP*	*nub-Gal4, UAS-ft, UAS-faf* $^{RNAi\ 55GD}$	<0.0001; ***
1j	*nub-Gal4, UAS-ft, UAS-GFP*	*nub-Gal4, UAS-ft, UAS-faf* $^{RNAi\ KK}$	<0.0001; ***
1j	*nub-Gal4, UAS-ft, UAS-GFP*	*nub-Gal4, UAS-ft, UAS-faf* $^{RNAi\ 79GD}$	<0.0001; ***
1j	*nub-Gal4, UAS-ft, UAS-GFP*	*nub-Gal4, UAS-ft, UAS-faf* ^{isoC}	<0.0001; ***
1j	*nub-Gal4, UAS-ft* ^{RNAi}*, UAS-GFP*	*nub-Gal4, UAS-ft* ^{RNAi}*, UAS-faf* $^{RNAi\ 55GD}$	<0.0001; ***
1j	*nub-Gal4, UAS-ft* ^{RNAi}*, UAS-GFP*	*nub-Gal4, UAS-ft* ^{RNAi}*, UAS-faf* ^{isoC}	0.294; ns
1k	*nub-Gal4, UAS-GFP*	*nub-Gal4, UAS-faf* $^{RNAi\ 55GD}$	0.6243; ns
1k	*nub-Gal4, UAS-GFP*	*nub-Gal4, UAS-faf* $^{RNAi\ KK}$	0.5448; ns
1k	*nub-Gal4, UAS-GFP*	*nub-Gal4, UAS-faf* $^{RNAi\ 79GD}$	>0.9999; ns
1k	*nub-Gal4, UAS-GFP*	*nub-Gal4, UAS-faf* ^{isoC}	>0.9999; ns
1k	*nub-Gal4, UAS-GFP*	*nub-Gal4, UAS-ft*	0.0001; ***
1k	*nub-Gal4, UAS-GFP*	*nub-Gal4, UAS-ft, UAS-faf* $^{RNAi\ 55GD}$	<0.0001; ***
1k	*nub-Gal4, UAS-GFP*	*nub-Gal4, UAS-ft, UAS-faf* $^{RNAi\ KK}$	<0.0001; ***
1k	*nub-Gal4, UAS-GFP*	*nub-Gal4, UAS-ft, UAS-faf* $^{RNAi\ 79GD}$	<0.0001; ***
1k	*nub-Gal4, UAS-GFP*	*nub-Gal4, UAS-ft, UAS-faf* ^{isoC}	0.0004; ***
1k	*nub-Gal4, UAS-GFP*	*nub-Gal4, UAS-ft* ^{RNAi}	<0.0001; ***
1k	*nub-Gal4, UAS-GFP*	*nub-Gal4, UAS-ft* ^{RNAi}*, UAS-faf* $^{RNAi\ 55GD}$	<0.0001; ***
1k	*nub-Gal4, UAS-GFP*	*nub-Gal4, UAS-ft* ^{RNAi}*, UAS-faf* ^{isoC}	<0.0001; ***
1k	*nub-Gal4, UAS-ft, UAS-GFP*	*nub-Gal4, UAS-ft, UAS-faf* $^{RNAi\ 55GD}$	0.0821; ns
1k	*nub-Gal4, UAS-ft, UAS-GFP*	*nub-Gal4, UAS-ft, UAS-faf* $^{RNAi\ KK}$	>0.9999; ns
1k	*nub-Gal4, UAS-ft, UAS-GFP*	*nub-Gal4, UAS-ft, UAS-faf* $^{RNAi\ 79GD}$	0.2349; ns
1k	*nub-Gal4, UAS-ft, UAS-GFP*	*nub-Gal4, UAS-ft, UAS-faf* ^{isoC}	>0.9999; ns
1k	*nub-Gal4, UAS-ft* ^{RNAi}*, UAS-GFP*	*nub-Gal4, UAS-ft* ^{RNAi}*, UAS-faf* $^{RNAi\ 55GD}$	0.0112; *
1k	*nub-Gal4, UAS-ft* ^{RNAi}*, UAS-GFP*	*nub-Gal4, UAS-ft* ^{RNAi}*, UAS-faf* ^{isoC}	0.6735; ns
2h	w^{iso}	$ft^{G\text{-}rv} / ft^{8}$	<0.0001; ***
2h	w^{iso}	$ft^{G\text{-}rv} / ft^{8}$; faf^{F08} / +	<0.0001; ***
2h	w^{iso}	$ft^{G\text{-}rv} / ft^{8}$; faf^{BX4} / +	<0.0001; ***
2h	w^{iso}	$ft^{G\text{-}rv} / ft^{8}$; faf^{B3} / +	<0.0001; ***
2h	w^{iso}	$ft^{G\text{-}rv} / ft^{8}$; faf^{BX3} / +	<0.0001; ***
2h	$ft^{G\text{-}rv} / ft^{8}$	$ft^{G\text{-}rv} / ft^{8}$; faf^{F08} / +	<0.0001; ***

2h	$ft^{G\text{-}rv}$ / ft^{8}	$ft^{G\text{-}rv}$ / ft^{8}; faf^{BX4} / +	0.0254; *
2h	$ft^{G\text{-}rv}$ / ft^{8}	$ft^{G\text{-}rv}$ / ft^{8}; faf^{B3} / +	0.0051; **
2h	$ft^{G\text{-}rv}$ / ft^{8}	$ft^{G\text{-}rv}$ / ft^{8}; faf^{BX3} / +	0.0054; **
3p	*nub-Gal4, UAS-GFP*	*nub-Gal4, UAS-Ds, UAS-GFP*	<0.0001; ***
3p	*nub-Gal4, UAS-GFP*	*nub-Gal4, UAS-Ds, UAS-*$faf^{RNAi\ 79GD}$	<0.0001; ***
3p	*nub-Gal4, UAS-GFP*	*nub-Gal4, UAS-Ds, UAS-*$faf^{RNAi\ KK}$	0.0002; ***
3p	*nub-Gal4, UAS-GFP*	*nub-Gal4, UAS-Ds, UAS-*$faf^{RNAi\ 55GD}$	<0.0001; ***
3p	*nub-Gal4, UAS-GFP*	*nub-Gal4, UAS-Ds, UAS-*faf^{isoC}	<0.0001; ***
3p	*nub-Gal4, UAS-Ds, UAS-GFP*	*nub-Gal4, UAS-Ds, UAS-*$faf^{RNAi\ 79GD}$	0.0015; **
3p	*nub-Gal4, UAS-Ds, UAS-GFP*	*nub-Gal4, UAS-Ds, UAS-*$faf^{RNAi\ KK}$	<0.0001; ***
3p	*nub-Gal4, UAS-Ds, UAS-GFP*	*nub-Gal4, UAS-Ds, UAS-*$faf^{RNAi\ 55GD}$	<0.0001; ***
3p	*nub-Gal4, UAS-Ds, UAS-GFP*	*nub-Gal4, UAS-Ds, UAS-*faf^{isoC}	<0.0001; ***
3q	*nub-Gal4, UAS-GFP*	*nub-Gal4, UAS-*$Dlish^{RNAi}$*, UAS-GFP*	<0.0001; ***
3q	*nub-Gal4, UAS-GFP*	*nub-Gal4, UAS-*$Dlish^{RNAi}$*, UAS-*$faf^{RNAi\ 55GD}$	0.962; ns
3q	*nub-Gal4, UAS-GFP*	*nub-Gal4, UAS-*$Dlish^{RNAi}$*, UAS-*faf^{isoC}	<0.0001; ***
3q	*nub-Gal4, UAS-GFP*	*nub-Gal4, UAS-*$Dlish^{RNAi}$*, UAS-ft*	<0.0001; ***
3q	*nub-Gal4, UAS-GFP*	*nub-Gal4, UAS-*$Dlish^{RNAi}$*, UAS-ft; UAS-*$faf^{RNAi\ 55GD}$	<0.0001; ***
3q	*nub-Gal4, UAS-*$Dlish^{RNAi}$*, UAS-GFP*	*nub-Gal4, UAS-*$Dlish^{RNAi}$*, UAS-*$faf^{RNAi\ 55GD}$	<0.0001; ***
3q	*nub-Gal4, UAS-*$Dlish^{RNAi}$*, UAS-GFP*	*nub-Gal4, UAS-*$Dlish^{RNAi}$*, UAS-*faf^{isoC}	<0.0001; ***
3q	*nub-Gal4, UAS-*$Dlish^{RNAi}$*, UAS-GFP*	*nub-Gal4, UAS-*$Dlish^{RNAi}$*, UAS-ft*	<0.0001; ***
3q	*nub-Gal4, UAS-*$Dlish^{RNAi}$*, UAS-GFP*	*nub-Gal4, UAS-*$Dlish^{RNAi}$*, UAS-ft; UAS-*$faf^{RNAi\ 55GD}$	<0.0001; ***
3r	*nub-Gal4, UAS-GFP*	*nub-Gal4, UAS-Fbxl7, UAS-GFP*	<0.0001; ***
3r	*nub-Gal4, UAS-GFP*	*nub-Gal4, UAS-Fbxl7, UAS-*$faf^{RNAi\ 55GD}$	<0.0001; ***
3r	*nub-Gal4, UAS-GFP*	*nub-Gal4, UAS-Fbxl7, UAS-ft*	<0.0001; ***
3r	*nub-Gal4, UAS-GFP*	*nub-Gal4, UAS-Fbxl7, UAS-ft, UAS-*$faf^{RNAi\ 55GD}$	<0.0001; ***
3r	*nub-Gal4, UAS-Fbxl7, UAS-GFP*	*nub-Gal4, UAS-Fbxl7, UAS-*$faf^{RNAi\ 55GD}$	0.3877; ns
5f	*en-Gal4, UAS-GFP*	*en-Gal4, UAS-*hpo^{RNAi}	0.0003; ***
5f	*en-Gal4, UAS-GFP*	*en-Gal4, UAS-*faf^{RNAi}	0.0521; ns
5f	*en-Gal4, UAS-GFP*	*en-Gal4, UAS-ft*	<0.0001; ***
5f	*en-Gal4, UAS-GFP*	*en-Gal4, UAS-ft, UAS-*faf^{RNAi}	0.0182; *
5f	*en-Gal4, UAS-ft*	*en-Gal4, UAS-ft, UAS-*faf^{RNAi}	<0.0001; ***
5l	*en-Gal4, UAS-*$lacZ^{RNAi}$	*en-Gal4, UAS-*hpo^{RNAi}	<0.0001; ***
5l	*en-Gal4, UAS-*$lacZ^{RNAi}$	*en-Gal4, UAS-*faf^{RNAi}	<0.0001; ***
5l	*en-Gal4, UAS-*$lacZ^{RNAi}$	*en-Gal4, UAS-ft*	0.0082; **
5l	*en-Gal4, UAS-*$lacZ^{RNAi}$	*en-Gal4, UAS-ft, UAS-*faf^{RNAi}	0.7406; ns
5l	*en-Gal4, UAS-ft*	*en-Gal4, UAS-ft, UAS-*faf^{RNAi}	<0.0001; ***
6e	*en-Gal4, UAS-GFP*	*en-Gal4, UAS-*faf^{isoC}	0.0287; *
6e	*en-Gal4, UAS-GFP*	*en-Gal4, UAS-*faf^{WT}	<0.0001; ***
6e	*en-Gal4, UAS-GFP*	*en-Gal4, UAS-*faf^{CD}	0.9979; ns
6j	*nub-Gal4, UAS-GFP*	*nub-Gal4, UAS-*faf^{isoC}	<0.0001; ***
6j	*nub-Gal4, UAS-GFP*	*nub-Gal4, UAS-*faf^{WT}	<0.0001; ***
6j	*nub-Gal4, UAS-GFP*	*nub-Gal4, UAS-*faf^{CD}	0.0148; *
6o	*nub-Gal4, UAS-GFP*	*nub-Gal4, UAS-ft*	<0.0001; ***
6o	*nub-Gal4, UAS-GFP*	*nub-Gal4, UAS-ft, UAS-*faf^{isoC}	<0.0001; ***
6o	*nub-Gal4, UAS-GFP*	*nub-Gal4, UAS-ft, UAS-*faf^{WT}	<0.0001; ***
6o	*nub-Gal4, UAS-GFP*	*nub-Gal4, UAS-ft, UAS-*faf^{CD}	<0.0001; ***

6o	*nub-Gal4, UAS-ft, UAS-GFP*	*nub-Gal4, UAS-ft, UAS-faf* ^{isoC}	<0.0001; ***
6o	*nub-Gal4, UAS-ft, UAS-GFP*	*nub-Gal4, UAS-ft, UAS-faf* ^{WT}	<0.0001; ***
6o	*nub-Gal4, UAS-ft, UAS-GFP*	*nub-Gal4, UAS-ft, UAS-faf* ^{CD}	0.0376; *
6t	*nub-Gal4, UAS-GFP*	*nub-Gal4, UAS-ft*^{RNAi}	0.0025; **
6t	*nub-Gal4, UAS-GFP*	*nub-Gal4, UAS-ft*^{RNAi}*, UAS-faf* ^{isoC}	0.6916; ns
6t	*nub-Gal4, UAS-GFP*	*nub-Gal4, UAS-ft*^{RNAi}*, UAS-faf* ^{WT}	<0.0001; ***
6t	*nub-Gal4, UAS-GFP*	*nub-Gal4, UAS-ft*^{RNAi}*, UAS-faf* ^{CD}	<0.0001; ***
6t	*nub-Gal4, UAS-ft*^{RNAi}*, UAS-GFP*	*nub-Gal4, UAS-ft*^{RNAi}*, UAS-faf* ^{isoC}	<0.0001; ***
6t	*nub-Gal4, UAS-ft*^{RNAi}*, UAS-GFP*	*nub-Gal4, UAS-ft*^{RNAi}*, UAS-faf* ^{WT}	<0.0001; ***
6t	*nub-Gal4, UAS-ft*^{RNAi}*, UAS-GFP*	*nub-Gal4, UAS-ft*^{RNAi}*, UAS-faf* ^{CD}	0.3518; ns
7d	*hh-Gal4, UAS-lacZ*^{RNAi}	*hh-Gal4, UAS-faf* ^{isoC}	<0.0001; ***
7d	*hh-Gal4, UAS-lacZ*^{RNAi}	*hh-Gal4, UAS-faf* ^{WT}	<0.0001; ***
7d	*hh-Gal4, UAS-lacZ*^{RNAi}	*hh-Gal4, UAS-faf* ^{CD}	0.9982; ns
7e	*hh-Gal4, UAS-lacZ*^{RNAi}	*hh-Gal4, UAS-faf* ^{isoC}	0.6521; ns
7e	*hh-Gal4, UAS-lacZ*^{RNAi}	*hh-Gal4, UAS-faf* ^{WT}	0.3803; ns
7e	*hh-Gal4, UAS-lacZ*^{RNAi}	*hh-Gal4, UAS-faf* ^{CD}	0.6248; ns
7f	*hh-Gal4, UAS-lacZ*^{RNAi}	*hh-Gal4, UAS-faf* ^{isoC}	<0.0001; ***
7f	*hh-Gal4, UAS-lacZ*^{RNAi}	*hh-Gal4, UAS-faf* ^{WT}	<0.0001; ***
7f	*hh-Gal4, UAS-lacZ*^{RNAi}	*hh-Gal4, UAS-faf* ^{CD}	0.9148; ns
7h	*Ø*	*faf* ^{WT}	0.0013; **
7h	*Ø*	*faf* ^{CD}	0.7596; ns
7h	*faf* ^{WT}	*faf* ^{CD}	0.0111; *
7j	*DMSO*	*WP1130*	0.0136; *
8e	*en-Gal4, UAS-GFP*	*en-Gal4, UAS-USP9X*	<0.0001; ***
8f	*en-Gal4, UAS-lacZ*^{RNAi}	*en-Gal4, UAS-USP9X*	<0.0001; ***
8i	*nub-Gal4, UAS-GFP*	*nub-Gal4, UAS-USP9X*	<0.0001; ***
8l	*nub-Gal4, UAS-GFP*	*nub-Gal4, UAS-ft*	<0.0001; ***
8l	*nub-Gal4, UAS-GFP*	*nub-Gal4, UAS-ft, UAS-USP9X*	<0.0001; ***
8l	*nub-Gal4, UAS-ft, UAS-GFP*	*nub-Gal4, UAS-ft, UAS-USP9X*	<0.0001; ***
8o	*nub-Gal4, UAS-GFP*	*nub-Gal4, UAS-ft*^{RNAi}	0.024; **
8o	*nub-Gal4, UAS-GFP*	*nub-Gal4, UAS-ft*^{RNAi}*, UAS-USP9X*	0.1459; ns
8o	*nub-Gal4, UAS-ft*^{RNAi}*, UAS-GFP*	*nub-Gal4, UAS-ft*^{RNAi}*, UAS-USP9X*	<0.0001; ***
9c	*hh-Gal4, UAS-lacZ*^{RNAi}	*hh-Gal4, UAS-USP9X*	0.0002; ***
9d	*hh-Gal4, UAS-lacZ*^{RNAi}	*hh-Gal4, UAS-USP9X*	0.9639; ns
9e	*hh-Gal4, UAS-lacZ*^{RNAi}	*hh-Gal4, UAS-USP9X*	<0.0001; ***
9g	*ø*	*USP9X*	0.044; *
9j	*GFP negative*	*GFP positive*	<0.0001; ***

Statistical analyses related to main Figures of the study. *, p<0.05; **, p<0.01; ***, p<0.001; ns, non-significant.

Figure S6a Source Data

Anti-HA

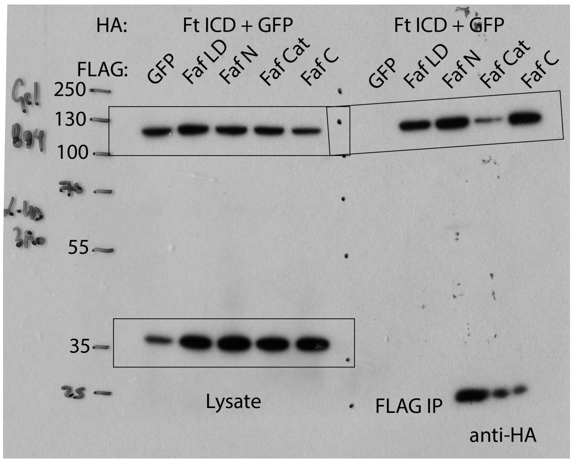

Anti-FLAG

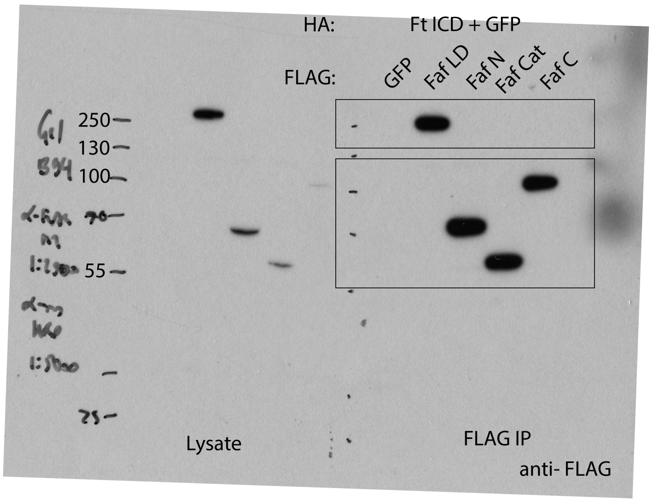

Anti-FLAG

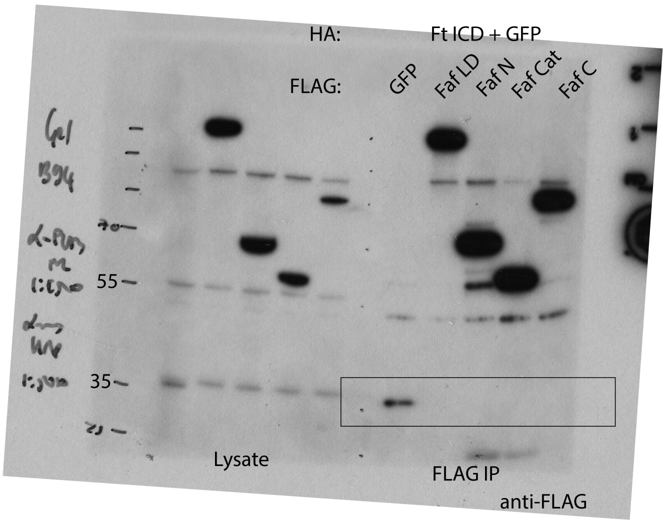

Anti-Tubulin

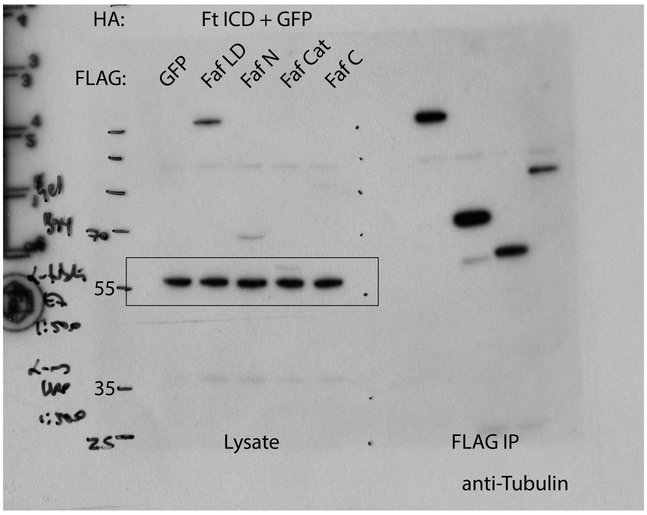

Figure S6d Source Data

Anti-FLAG

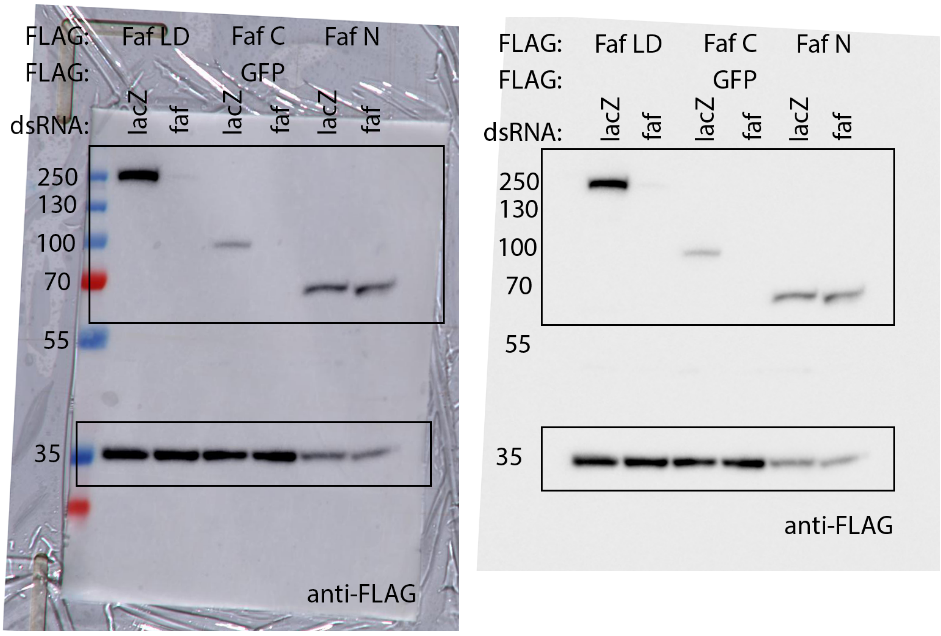

Anti-Tubulin

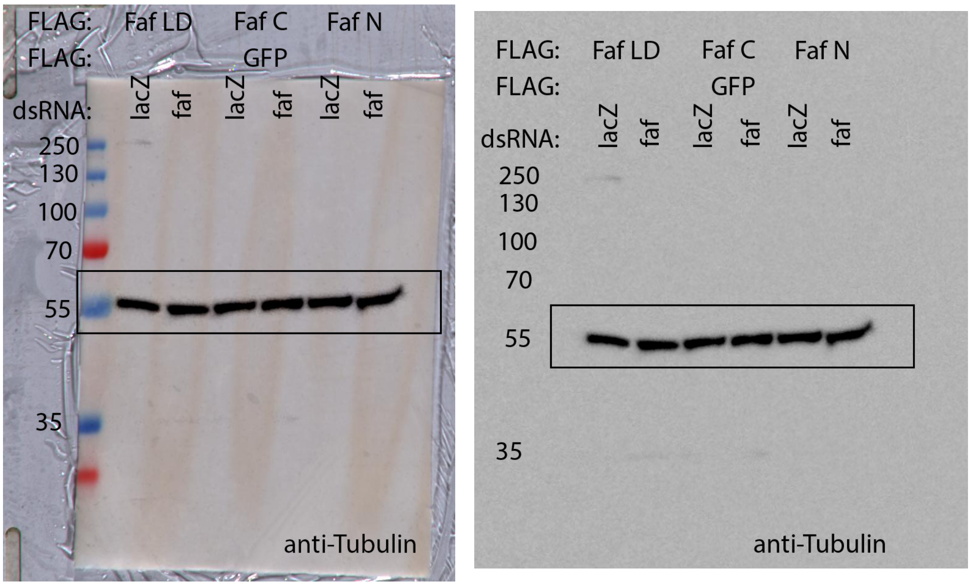

Figure S10a Source Data

Anti-HA

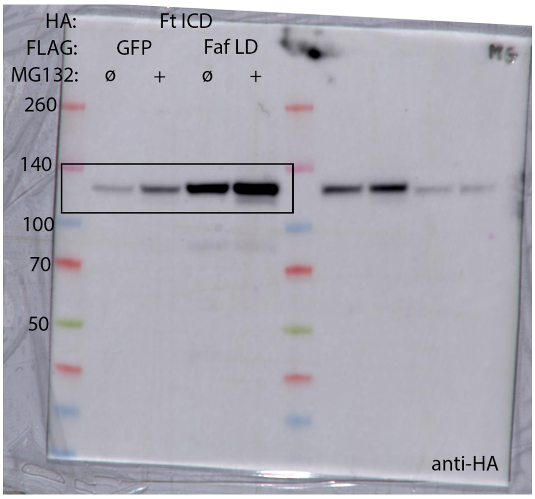

Anti-FLAG

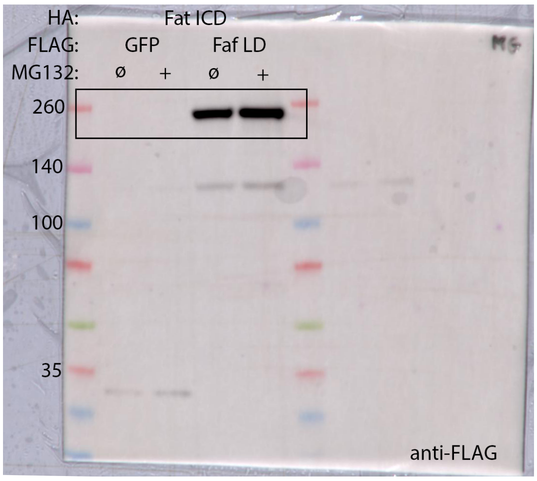

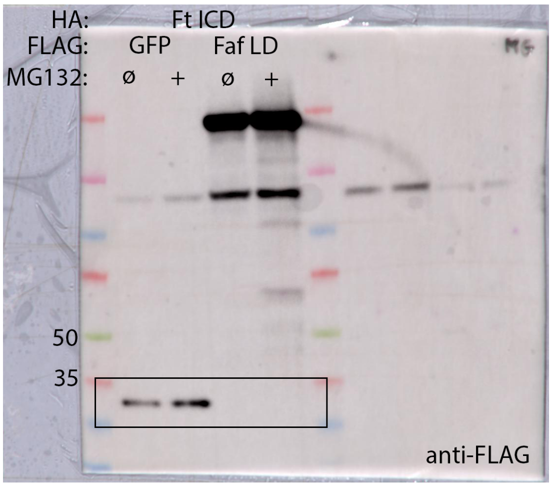

Anti-Tubulin

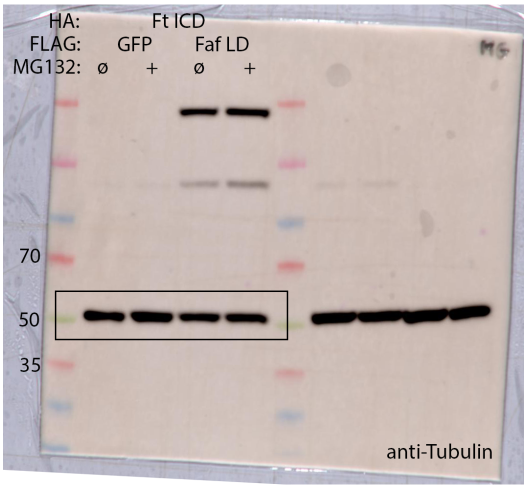

Figure S10b Source Data

Anti-HA

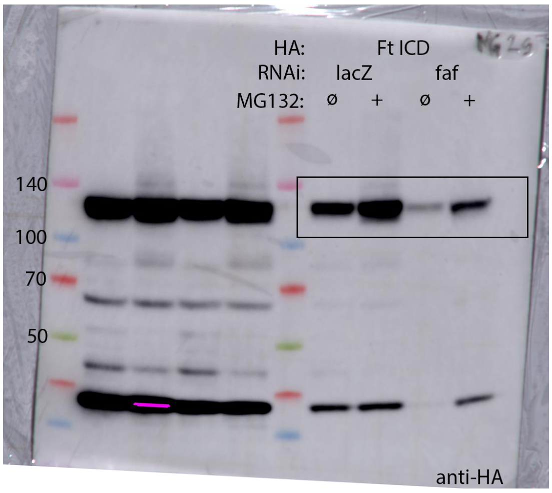

Anti-Tubulin

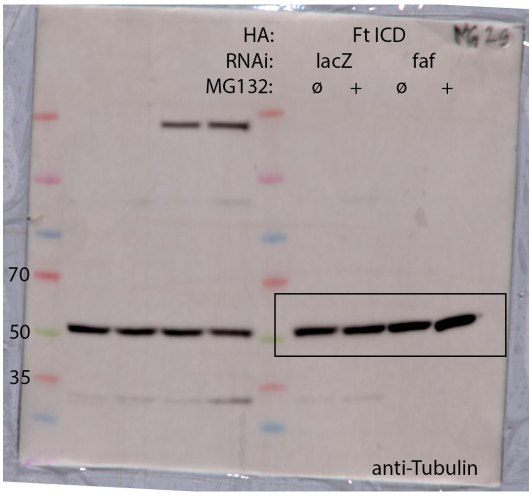

Figure S10c Source Data

Anti-HA

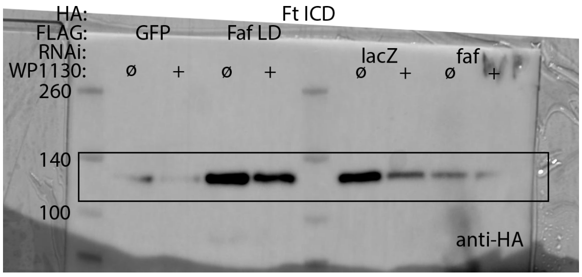

Anti-FLAG

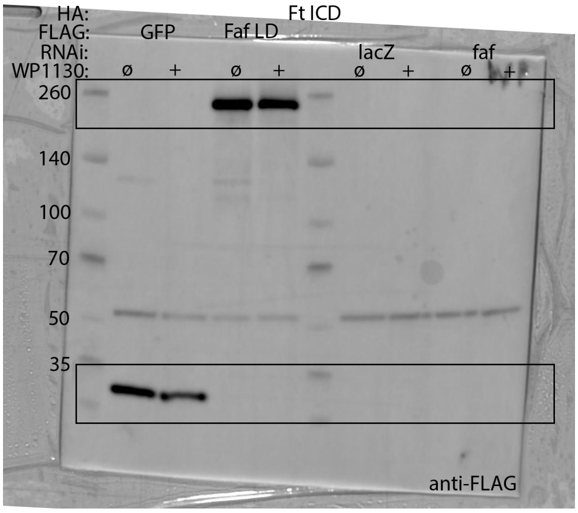

Anti-Tubulin

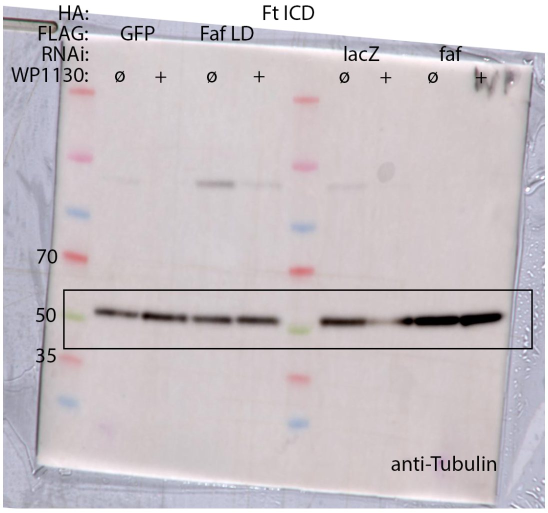

Figure S10h Source Data

Anti-FLAG

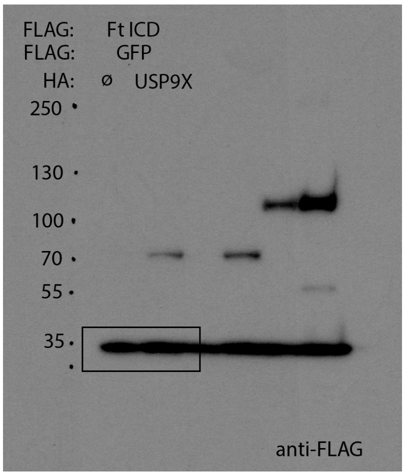

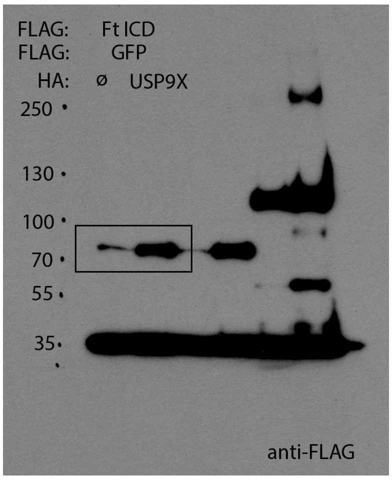

Anti-HA

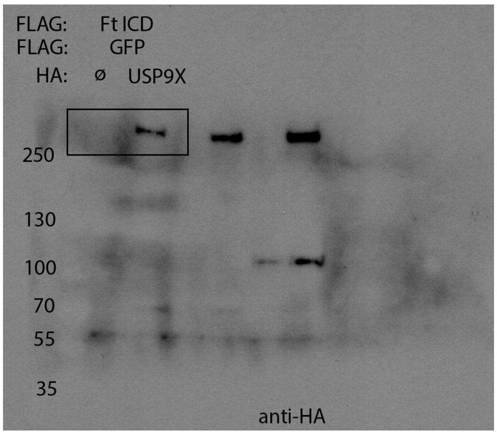

Anti-Tubulin

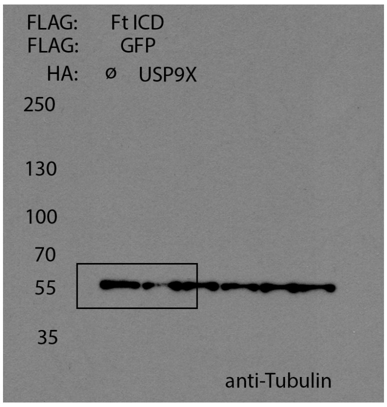

Supplement: Supplementary file 1 — Supplementary Information [file 41467_2025_57164_MOESM1_ESM.pdf]
