# Supplementary material for: The deubiquitylating enzyme Fat facets promotes Fat signalling and restricts tissue growth
Source: Nat Commun. 2025 Feb 24;16:1938. doi: 10.1038/s41467-025-57164-3 (PMC11850632; doi:10.1038/s41467-025-57164-3)
Supplement: Supplementary file 2 — Reporting Summary [file 41467_2025_57164_MOESM2_ESM.pdf]

nature portfolio

Corresponding author(s): Paulo S Ribeiro - NCOMMS-24-05779

Last updated by author(s): Dec 20, 2024

Reporting Summary

Nature Portfolio wishes to improve the reproducibility of the work that we publish. This form provides structure for consistency and transparency in reporting. For further information on Nature Portfolio policies, see our [Editorial Policies] and the [Editorial Policy Checklist].

Statistics

For all statistical analyses, confirm that the following items are present in the figure legend, table legend, main text, or Methods section.

n/a	Confirmed	
☐	☒	The exact sample size (*n*) for each experimental group/condition, given as a discrete number and unit of measurement
☐	☒	A statement on whether measurements were taken from distinct samples or whether the same sample was measured repeatedly
☐	☒	The statistical test(s) used AND whether they are one- or two-sided<br>*Only common tests should be described solely by name; describe more complex techniques in the Methods section.*
☐	☒	A description of all covariates tested
☐	☒	A description of any assumptions or corrections, such as tests of normality and adjustment for multiple comparisons
☐	☒	A full description of the statistical parameters including central tendency (e.g. means) or other basic estimates (e.g. regression coefficient) AND variation (e.g. standard deviation) or associated estimates of uncertainty (e.g. confidence intervals)
☐	☒	For null hypothesis testing, the test statistic (e.g. *F*, *t*, *r*) with confidence intervals, effect sizes, degrees of freedom and *P* value noted<br>*Give P values as exact values whenever suitable.*
☒	☐	For Bayesian analysis, information on the choice of priors and Markov chain Monte Carlo settings
☒	☐	For hierarchical and complex designs, identification of the appropriate level for tests and full reporting of outcomes
☒	☐	Estimates of effect sizes (e.g. Cohen's *d*, Pearson's *r*), indicating how they were calculated

Our web collection on statistics for biologists contains articles on many of the points above.

Software and code

Policy information about availability of computer code

Data collection	Data was collected using community standards for the following software: ZEN, Pannoramic Viewer
Data analysis	Data was analysed using community standards for the following software: ImageJ (1.53k) , Fiji (2.14.0), R Studio, Microsoft Excel (16.93.1), GraphPad Prism (8, 9 and 10), Ilastik, Adobe Photoshop (2025)

For manuscripts utilizing custom algorithms or software that are central to the research but not yet described in published literature, software must be made available to editors and reviewers. We strongly encourage code deposition in a community repository (e.g. GitHub). See the Nature Portfolio guidelines for submitting code & software for further information.

Data

Policy information about availability of data

All manuscripts must include a data availability statement. This statement should provide the following information, where applicable:

- Accession codes, unique identifiers, or web links for publicly available datasets
- A description of any restrictions on data availability
- For clinical datasets or third party data, please ensure that the statement adheres to our policy

Source data are provided with this paper.

Research involving human participants, their data, or biological material

Policy information about studies with human participants or human data. See also policy information about sex, gender (identity/presentation), and sexual orientation and race, ethnicity and racism.

Reporting on sex and gender	N/A
Reporting on race, ethnicity, or other socially relevant groupings	N/A
Population characteristics	N/A
Recruitment	N/A
Ethics oversight	N/A

Note that full information on the approval of the study protocol must also be provided in the manuscript.

Field-specific reporting

Please select the one below that is the best fit for your research. If you are not sure, read the appropriate sections before making your selection.

☒ Life sciences ☐ Behavioural & social sciences ☐ Ecological, evolutionary & environmental sciences

For a reference copy of the document with all sections, see nature.com/documents/nr-reporting-summary-flat.pdf

Life sciences study design

All studies must disclose on these points even when the disclosure is negative.

Sample size	Sample sizes were determined according to previous research using similar in vivo approaches. Sample sizes were not predetermined or calculated beforehand. In all experiments, sample size is equal to or over 8 experimental animals for in vivo approaches. For in vitro experiments, sample sizes were equal to or over 3 independent experiments
Data exclusions	N/A
Replication	All in vitro experiments are n=3 or n>3 independent experiments to ensure reproducibility
Randomization	Majority of in vivo data included randomisation of animals used for analysis within the same sample
Blinding	Majority of in vivo data was analysed blinded. Different genotype groups were assigned specific codes associated with a particular experimental repeat and data was only deanonymised after analysis was completed

Reporting for specific materials, systems and methods

We require information from authors about some types of materials, experimental systems and methods used in many studies. Here, indicate whether each material, system or method listed is relevant to your study. If you are not sure if a list item applies to your research, read the appropriate section before selecting a response.

Materials & experimental systems

n/a	Involved in the study	
☐	☒	Antibodies
☐	☒	Eukaryotic cell lines
☒	☐	Palaeontology and archaeology
☐	☒	Animals and other organisms
☒	☐	Clinical data
☒	☐	Dual use research of concern
☒	☐	Plants

Methods

n/a	Involved in the study	
☒	☐	ChIP-seq
☒	☐	Flow cytometry
☒	☐	MRI-based neuroimaging

Antibodies

Antibodies used	Mouse anti-FLAG (M2; Sigma; RRID:AB_262044), Rat anti-HA (3F10; Roche Applied Science; RRID:AB_2314622), Mouse anti-V5 (Thermo Fisher Scientific; RRID:AB_2556564), Mouse anti-tubulin (E7; DSHB; RRID:AB_528499). Mouse anti-Armadillo (N2 7A1; DSHB; RRID:AB_528089), Rat anti-Fat (kind gift from Helen McNeill), Mouse anti-β-galactosidase (Z3781, Promega; RRID:AB_430877), Rabbit anti-Fat4 (PA5-116735; Thermo Fisher Scientific; RRID: AB_2901366), HRP-conjugated sheep anti-mouse (Amersham) and

	HRP-conjugated goat anti-rat (Thermo Fisher Scientific), Anti-mouse Rhodamine Red-X-conjugated (Jackson ImmunoResearch), Anti-mouse Alexa Fluor 647-conjugated (Jackson ImmunoResearch), Anti-rat Alexa Fluor 647-conjugated (Jackson ImmunoResearch), Donkey anti-rabbit Rhodamine Red X-conjugated (Jackson ImmunoResearch)
Validation	All antibodies have been previously validated

Eukaryotic cell lines

Policy information about cell lines and Sex and Gender in Research

Cell line source(s)	HEK293 cells (RRID:CVCL_0045) were obtained from the ATCC
Authentication	Cell lines were previously STR authenticated
Mycoplasma contamination	Cell lines were tested for mycoplasma contamination and were found not to have any contamination
Commonly misidentified lines (See ICLAC register)	No commonly misindentified lines have been used

Animals and other research organisms

Policy information about studies involving animals; ARRIVE guidelines recommended for reporting animal research, and Sex and Gender in Research

Laboratory animals	Drosophila melanogaster; animals were used at the larval and adult stages
Wild animals	N/A
Reporting on sex	Most in vivo experiments used animals from both sexes, unless the genetic cross schemes resulted in the progeny of interest being of a single sex
Field-collected samples	N/A
Ethics oversight	No ethical approval required

Note that full information on the approval of the study protocol must also be provided in the manuscript.

Plants

Seed stocks	N/A
Novel plant genotypes	N/A
Authentication	N/A
